# Supplementary material for: Antitumour activity of neratinib in patients with HER2-mutant advanced biliary tract cancers
Source: Nat Commun. 2023 Feb 6;14:630. doi: 10.1038/s41467-023-36399-y (PMC9902444; doi:10.1038/s41467-023-36399-y)
Supplement: Supplementary file 1 — Supplementary information [file 41467_2023_36399_MOESM1_ESM.pdf]

Supplementary information

Supplementary Table 1 Central confirmation of *HER2* mutations.

Patient ID	Enrolment assay	*HER2* mutation code	Confirmed by *HER2* targeted sequencing (Custom-seq)	Confirmed by MSK-IMPACT	Confirmed by MSK-ACCESS	Additional *HER2* mutations found	Best objective response
1	MDA Solid Tumor Genomic Assay	V777L	NA	NA	V777L		NE
2	Brigham and Women's in-house	L755S	NA	L755S	NA		SD
3	Guardant360	V777L	NA	V777L[a]	NA		PR
4	SNaPshot	V777L	NA	NA	NA		PD
5	FoundationOne	L755S	NA	NA	NA		PD
6	FoundationOne	G776C; H878Y	NA	NA	G776C; H878Y		SD
7	Guardant360	S310F	NA	NA	S310F[a]	D277Y	PD
8	Caris MiProfile	D769Y	NA	NA	D769Y		SD
9	FoundationOne	S310F	NA	NA	S310F		PD
10	FoundationOne	S310F	NA	NA	S310F		NE
11	Pangaea Sanger exon 8, 19−21	S310F	NA	S310F	NA		PD
12	RT-PCR	V842I	Inconclusive	Not confirmed	NA		NE
13	Oncomine	V842I	NA	V842I	NA		NE
14	FoundationOne	T733I	NA	T733I	NA		SD
15	MSK-IMPACT	S310F	S310F	S310F	NA		PR
16	MSK-IMPACT	S310F	S310F	S310F	NA		PD
17	MSK-IMPACT	S310Y	NA	S310Y	NA		SD
18	MSK-IMPACT	S310Y	NA	S310Y	NA		SD
19	Peter Mac somatic panel (tissue)	V659E	V695E	V695E	NA	V842I	uPR
20	AmpliSeq comprehensive cancer panel	S310F	NA	NA	S310F	L253V[a]	SD
21	AmpliSeq colon and lung cancer panel	V777L	NA	NA	V777L		PR
22	FoundationOne CDx	R678Q	NA	R678Q	NA		NE
23	FoundationOne CDx	S310F; V777L	NA	S310F; V777L	NA		PD
24	FoundationOne	R678Q	NA	R678Q	NA		NE
25	Stanford STAMP	S310F	NA	S310F	NA		PR

[a]Alteration at or near limit of detection of the assay, underwent manual re-review to confirm accuracy of alteration.

MDA MD Anderson, *MSK-ACCESS* Memorial Sloan Kettering-Analysis of Circulating cfDNA to Evaluate Somatic Status, *MSK-IMPACT* Integrated Mutation Profiling of Actionable Cancer Targets, *NA* not available, *NE* not evaluable, *PD* progressive disease, *PR* partial response, *RT-PCR* reverse transcription polymerase chain reaction, *SD* stable disease, *STAMP* Solid Tumor Actionable Mutation Panel, *uPR* unconfirmed partial response.

Supplementary Table 2 Serious adverse events.

Adverse event	Biliary tract (n=25)
Patients with ≥1 reported serious adverse event	16 (64%)
Dehydration	3 (12%)
Abdominal pain	2 (8%)
Diarrhoea	2 (8%)
Pyrexia	2 (8%)
Sepsis	2 (8%)
Acute kidney injury	1 (4%)
Anaemia	1 (4%)
Asthenia	1 (4%)
Bacteraemia	1 (4%)
Bile duct stone	1 (4%)
Blood bilirubin increased	1 (4%)
Decreased appetite	1 (4%)
Device-related infection	1 (4%)
Encephalopathy	1 (4%)
Gallbladder obstruction	1 (4%)
Gastric ulcer	1 (4%)
General physical health deterioration	1 (4%)
Hypotension	1 (4%)
International normalised ratio increased	1 (4%)
Large intestinal haemorrhage	1 (4%)
Liver abscess	1 (4%)
Malnutrition	1 (4%)
Nausea	1 (4%)
Obstruction gastric	1 (4%)
Respiratory failure	1 (4%)
Small intestinal obstruction	1 (4%)
Vomiting	1 (4%)

Data are n (%).

Supplementary Table 3 Characteristics of diarrhoea.

Characteristic	Biliary tract cancer (n=25)
Diarrhoea incidence	
Any grade	14 (56%)
Grade 1	3 (12%)
Grade 2	5 (20%)
Grade 3	6 (24%)
Action taken for diarrhoea	
Concomitant medication	10 (40%)
Leading to temporary dose hold	3 (12%)
Leading to hospitalisation	1 (4%)
Diarrhoea episodes	
Median time to first grade 3 diarrhoea, days (range)	7 (3–28)
Median duration per episode of grade 3 diarrhoea, days (range)	5.5 (1–56)

Data are n (%) unless otherwise stated.

Supplementary Table 4 Participating institutions and ethics committees.

**Institution**	**Ethics Committee**
Memorial Sloan-Kettering Cancer Center, USA	Memorial Sloan-Kettering Cancer Center Institutional Review Board/Privacy Board
Dana-Farber Cancer Institute, USA	Dana-Farber Cancer Institute Institutional Review Board
Washington University in St. Louis, USA	The Washington University in St. Louis Institutional Review Board
The University of Texas MD Anderson Cancer Center, USA	MD Anderston Institutional Review Board
University of Southern California, USA	University of Southern California Institutional Review Board
Stanford University, USA	Administrative Panel on Human Subjects in Medical Research
Roswell Park Comprehensive Cancer Center, USA	Roswell Park Comprehensive Cancer Center Institutional Review Board
Mayo Clinic in Arizona, USA	Mayo Clinic Institutional Review Boards
CEIC Hospital Universitari Vall d'Hebron, Spain	Clinical Research Ethics Committee of "CEIC Hospital de Madrid"
CEIC USP Institut Universitari Dexeus, Spain	Clinical Research Ethics Committee of "CEIC Hospital de Madrid"
CEIC Fundación Jímenez Díaz, Spain	Clinical Research Ethics Committee of "CEIC Hospital de Madrid"
Institut Gustave Roussy, France	Nord-Ouest II Independent Ethics Committee
Centre Leon Berard, France	Nord-Ouest II Independent Ethics Committee
Peter MacCallum Cancer Centre, Australia	Peter MacCallum Cancer Centre Ethics Committee
Davidoff Cancer Center, Israel	Helsinki Committee Rabin Medical Center-Beilinson Hospital
University Hospital, Rigshospitalet, Denmark	The Captial Region of Denmark Health Centre Ethics Committee
